# Supplementary material for: Targeting ribosome biogenesis as a novel therapeutic approach to overcome EMT-related chemoresistance in breast cancer
Source: eLife. 2024 Sep 11;12:RP89486. doi: 10.7554/eLife.89486 (PMC11390108; doi:10.7554/eLife.89486)
Supplement: Figure 1—source data 1. [file elife-89486-fig1-data1.docx]

**Figure 1-source data 1:**

**EMT marker genes that were used in calculation of AUCell value of EMT status for Tri-PyMT cells:**

| **Epithelial marker genes:** | Cdh1, Cldn3, Cldn7, Epcam, Rfp, Krt8, Krt7, Krt18, Serpinb5, Wfdc2 |
| --- | --- |
| **Mesenchymal marker genes:** | Col6a1, Col6a2, Fn1, Gfp, Il33, Mgp, Pdgfrb, S100a4, Vim, Zeb1 |
