# Supplementary material for: Targeting ribosome biogenesis as a novel therapeutic approach to overcome EMT-related chemoresistance in breast cancer
Source: eLife. 2024 Sep 11;12:RP89486. doi: 10.7554/eLife.89486 (PMC11390108; doi:10.7554/eLife.89486)
Supplement: Figure 1—source data 2. [file elife-89486-fig1-data2.docx]

**Figure 1-source data 2:**

**Enriched pathways in GSEAs of EMT transitioning cells:**

|  |  | **Trans vs Epi** | | **Trans vs Mes** | |
| --- | --- | --- | --- | --- | --- |
| **PATHWAY NAME** | **SIZE** | **NES** | **NOM p-val** | **NES** | **NOM p-val** |
| GOBP_CELL_CELL_JUNCTION_ORGANIZATION | 123 | 1.570 | 0.008 | 2.450 | 0.000 |
| GOBP_SKIN_DEVELOPMENT | 170 | 1.760 | 0.000 | 2.270 | 0.000 |
| GOBP_EPIDERMIS_DEVELOPMENT | 194 | 1.690 | 0.000 | 2.240 | 0.000 |
| GOBP_KERATINOCYTE_DIFFERENTIATION | 70 | 1.730 | 0.000 | 2.210 | 0.000 |
| **GOBP_RIBOSOMAL_LARGE_SUBUNIT_BIOGENESIS** | 68 | 1.730 | 0.008 | 2.190 | 0.000 |
| **GOBP_RIBOSOME_BIOGENESIS** | 291 | 1.640 | 0.008 | 2.190 | 0.000 |
| **GOBP_RIBONUCLEOPROTEIN_COMPLEX_BIOGENESIS** | 392 | 1.630 | 0.010 | 2.180 | 0.000 |
| GOBP_EPIDERMAL_CELL_DIFFERENTIATION | 104 | 1.690 | 0.002 | 2.150 | 0.000 |
| **GOBP_RRNA_METABOLIC_PROCESS** | 233 | 1.750 | 0.004 | 2.150 | 0.000 |
| GOBP_RNA_PHOSPHODIESTER_BOND_HYDROLYSIS_ENDONUCLEOLYTIC | 26 | 1.830 | 0.000 | 2.040 | 0.002 |
| GOBP_EPITHELIAL_CELL_DIFFERENTIATION | 346 | 1.580 | 0.000 | 2.020 | 0.000 |
| GOBP_ACTIN_FILAMENT_BASED_MOVEMENT | 57 | 1.770 | 0.000 | 2.000 | 0.000 |
| GOBP_CELL_JUNCTION_ASSEMBLY | 232 | 1.850 | 0.000 | 1.980 | 0.000 |
| GOBP_CELL_CELL_ADHESION | 371 | 1.620 | 0.000 | 1.980 | 0.000 |
| **GOBP_ENDONUCLEOLYTIC_CLEAVAGE_INVOLVED_IN_RRNA_PROCESSING** | 16 | 1.890 | 0.000 | 1.920 | 0.006 |
| GOBP_RNA_LOCALIZATION | 143 | 1.690 | 0.006 | 1.900 | 0.000 |
| GOBP_POSITIVE_REGULATION_OF_MRNA_PROCESSING | 34 | 1.670 | 0.006 | 1.890 | 0.000 |
| GOBP_POSITIVE_REGULATION_OF_MRNA_METABOLIC_PROCESS | 112 | 1.730 | 0.002 | 1.880 | 0.000 |
| GOBP_ESTABLISHMENT_OR_MAINTENANCE_OF_CELL_POLARITY | 158 | 1.830 | 0.000 | 1.870 | 0.000 |
| GOBP_ESTABLISHMENT_OF_RNA_LOCALIZATION | 121 | 1.690 | 0.008 | 1.860 | 0.002 |
| GOBP_NUCLEAR_TRANSPORT | 281 | 1.700 | 0.000 | 1.850 | 0.000 |
| GOBP_REGULATION_OF_MRNA_METABOLIC_PROCESS | 236 | 1.590 | 0.010 | 1.830 | 0.000 |
| GOBP_TISSUE_MORPHOGENESIS | 401 | 1.600 | 0.000 | 1.820 | 0.000 |
| GOBP_ACTIN_MEDIATED_CELL_CONTRACTION | 39 | 1.770 | 0.002 | 1.820 | 0.002 |
| GOBP_MUSCLE_CONTRACTION | 118 | 1.610 | 0.008 | 1.820 | 0.002 |
| GOBP_NUCLEAR_EXPORT | 141 | 1.710 | 0.000 | 1.810 | 0.000 |
| GOBP_GLAND_MORPHOGENESIS | 95 | 1.560 | 0.000 | 1.810 | 0.000 |
| GOBP_MRNA_TRANSPORT | 95 | 1.710 | 0.006 | 1.810 | 0.002 |
| **GOBP_RIBOSOMAL_SUBUNIT_EXPORT_FROM_NUCLEUS** | 16 | 1.850 | 0.000 | 1.800 | 0.002 |
| GOBP_CELL_JUNCTION_ORGANIZATION | 428 | 1.700 | 0.000 | 1.800 | 0.000 |
| GOBP_ESTABLISHMENT_OF_CELL_POLARITY | 118 | 1.740 | 0.004 | 1.790 | 0.002 |
| GOBP_IMPORT_INTO_NUCLEUS | 140 | 1.590 | 0.008 | 1.790 | 0.000 |
| GOBP_POSITIVE_REGULATION_OF_LOCOMOTION | 366 | 1.660 | 0.000 | 1.760 | 0.000 |
| GOBP_REGULATION_OF_ERBB_SIGNALING_PATHWAY | 56 | 1.670 | 0.002 | 1.760 | 0.004 |
| GOBP_REGULATION_OF_CELL_ADHESION | 419 | 1.590 | 0.000 | 1.750 | 0.000 |
| GOBP_CELL_MATRIX_ADHESION | 127 | 1.820 | 0.002 | 1.750 | 0.002 |
| GOBP_NUCLEOBASE_CONTAINING_COMPOUND_TRANSPORT | 151 | 1.690 | 0.008 | 1.730 | 0.004 |
| GOBP_ACTIN_FILAMENT_BASED_PROCESS | 471 | 1.720 | 0.000 | 1.720 | 0.000 |
| GOBP_REGULATION_OF_NUCLEOCYTOPLASMIC_TRANSPORT | 104 | 1.750 | 0.000 | 1.710 | 0.008 |
| GOBP_PROTEIN_LOCALIZATION_TO_NUCLEUS | 253 | 1.640 | 0.004 | 1.680 | 0.000 |
| GOBP_ACTOMYOSIN_STRUCTURE_ORGANIZATION | 122 | 1.590 | 0.006 | 1.680 | 0.004 |
| GOBP_ERBB_SIGNALING_PATHWAY | 82 | 1.800 | 0.000 | 1.670 | 0.008 |
| GOBP_RNA_DESTABILIZATION | 78 | 1.690 | 0.006 | 1.670 | 0.004 |
| GOBP_CELL_SUBSTRATE_ADHESION | 200 | 1.710 | 0.002 | 1.660 | 0.002 |
| GOBP_REGULATION_OF_PROTEIN_LOCALIZATION_TO_NUCLEUS | 117 | 1.570 | 0.004 | 1.660 | 0.002 |
| GOBP_RNA_MEDIATED_GENE_SILENCING | 87 | 1.650 | 0.010 | 1.650 | 0.009 |
| GOBP_NUCLEUS_ORGANIZATION | 114 | 1.790 | 0.000 | 1.640 | 0.006 |
| GOBP_HEART_PROCESS | 108 | 1.690 | 0.000 | 1.600 | 0.004 |
| GOBP_MUSCLE_SYSTEM_PROCESS | 184 | 1.680 | 0.006 | 1.540 | 0.006 |

* GSEAs of scRNA-seq data were performed with BP subset of GO pathway (3185 gene sets with a filter of 15 – 500 genes/set). Totally, 49 gene sets were overlapped with the significantly enriched gene sets (P < 0.01) in comparisons of Trans vs Epi and Trans vs Mes. Gene set size (number of genes), normalized enrich scores (NES) and nominal P values (NOM p-val) are shown in the table. Pathways related to Ribosome or RRNA processing were highlighted in red.
