# Supplementary figures and images for: Targeting ribosome biogenesis as a novel therapeutic approach to overcome EMT-related chemoresistance in breast cancer
Source: eLife. 2024 Sep 11;12:RP89486. doi: 10.7554/eLife.89486 (PMC11390108; doi:10.7554/eLife.89486)

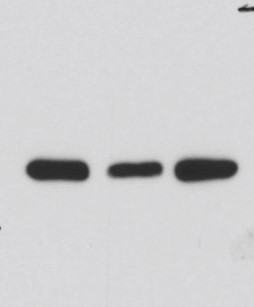

Supplement: Figure 1—source data 3. [file elife-89486-fig1-data3.zip › Fig1B/Fig1b_bactin.jpg]

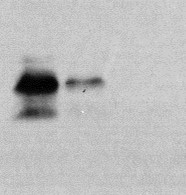

Supplement: Figure 1—source data 3. [file elife-89486-fig1-data3.zip › Fig1B/Fig1b_Ecad.jpg]

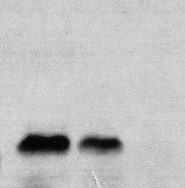

Supplement: Figure 1—source data 3. [file elife-89486-fig1-data3.zip › Fig1B/Fig1b_Epcam.jpg]

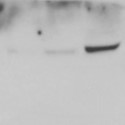

Supplement: Figure 1—source data 3. [file elife-89486-fig1-data3.zip › Fig1B/Fig1b_Vim.jpg]

**Figure 1B**, Western blot of EMT markers with flow sorted RFP+ (R), Doub+ (D), and GFP+ (G) Tri-PyMT cells.

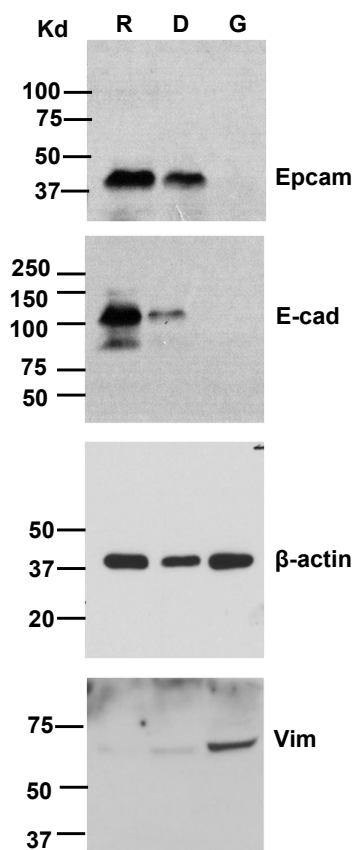

Supplement: Figure 1—source data 4. [file elife-89486-fig1-data4.pdf]

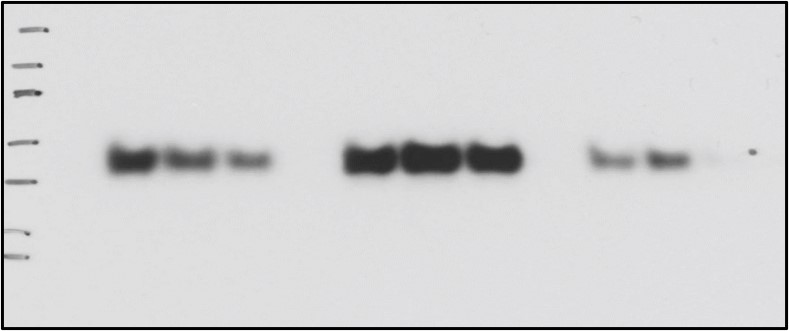

Supplement: Figure 3—source data 1. [file elife-89486-fig3-data1.zip › Fig3A/Fig3a_pERK.jpg]

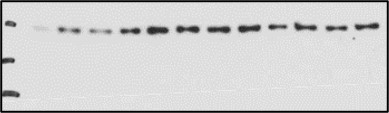

Supplement: Figure 3—source data 1. [file elife-89486-fig3-data1.zip › Fig3A/Fig3a_pmTOR.jpg]

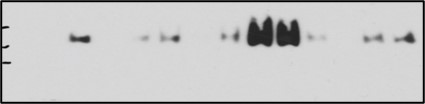

Supplement: Figure 3—source data 1. [file elife-89486-fig3-data1.zip › Fig3A/Fig3a_prpS6.jpg]

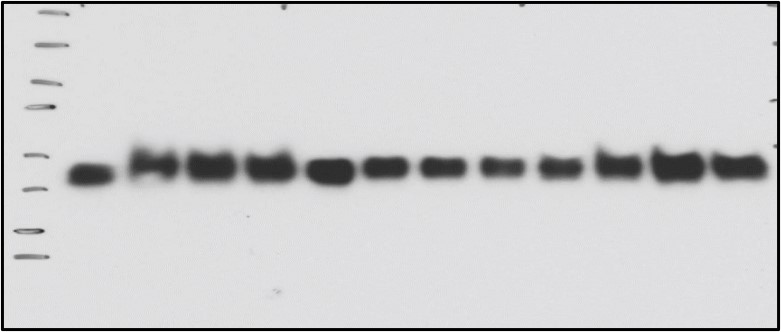

Supplement: Figure 3—source data 1. [file elife-89486-fig3-data1.zip › Fig3A/Fig3a_TotalERK.jpg]

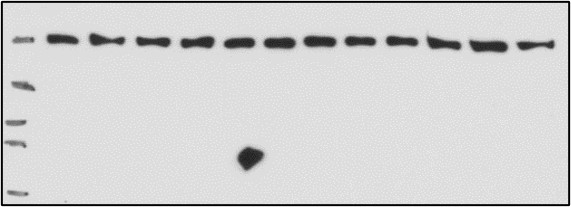

Supplement: Figure 3—source data 1. [file elife-89486-fig3-data1.zip › Fig3A/Fig3a_TotalmTOR.jpg]

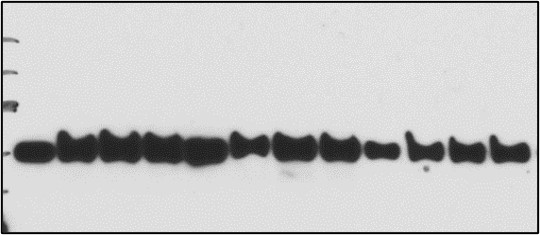

Supplement: Figure 3—source data 1. [file elife-89486-fig3-data1.zip › Fig3A/Fig3a_Tubulin.jpg]

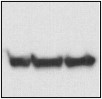

Supplement: Figure 4—source data 1. [file elife-89486-fig4-data1.zip › Fig4B/Fig4b_bactin.jpg]

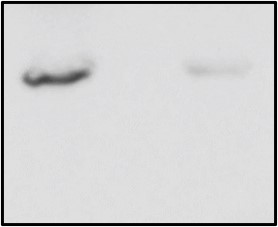

Supplement: Figure 4—source data 1. [file elife-89486-fig4-data1.zip › Fig4B/Fig4b_Snail.jpg]

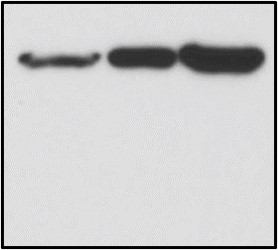

Supplement: Figure 4—source data 1. [file elife-89486-fig4-data1.zip › Fig4B/Fig4b_Ecad.jpg]

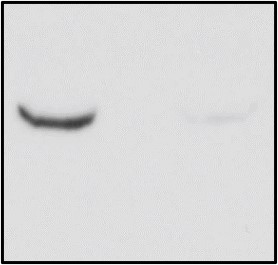

Supplement: Figure 4—source data 1. [file elife-89486-fig4-data1.zip › Fig4B/Fig4b_Vim.jpg]

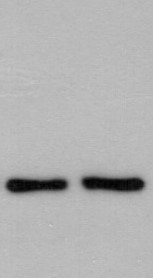

Supplement: Figure 4—figure supplement 2—source data 1. [file elife-89486-fig4-figsupp2-data1.zip › Fig4B_S2A/Fig4b_S2a_bactin28.jpg]

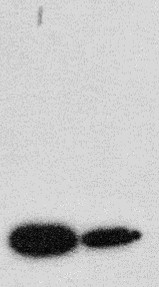

Supplement: Figure 4—figure supplement 2—source data 1. [file elife-89486-fig4-figsupp2-data1.zip › Fig4B_S2A/Fig4b_S2a_Rps24.jpg]

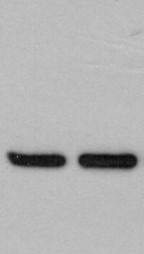

Supplement: Figure 4—figure supplement 2—source data 1. [file elife-89486-fig4-figsupp2-data1.zip › Fig4B_S2A/Fig4b_S2a_bactin.jpg]

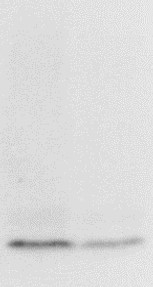

Supplement: Figure 4—figure supplement 2—source data 1. [file elife-89486-fig4-figsupp2-data1.zip › Fig4B_S2A/Fig4b_S2a_Rps28.jpg]
