# Supplementary material for: Targeting ribosome biogenesis as a novel therapeutic approach to overcome EMT-related chemoresistance in breast cancer
Source: eLife. 2024 Sep 11;12:RP89486. doi: 10.7554/eLife.89486 (PMC11390108; doi:10.7554/eLife.89486)

Figure 3A, Western blots show phospho-ERK, total ERK, phospho-mTORC1, total mTORC1 and p-rps6 in sorted RFP+, Doub+, and GFP+ Tri-PyMT cells following stimulation with 10% FBS at 0, 10, 30 and 60 mins.

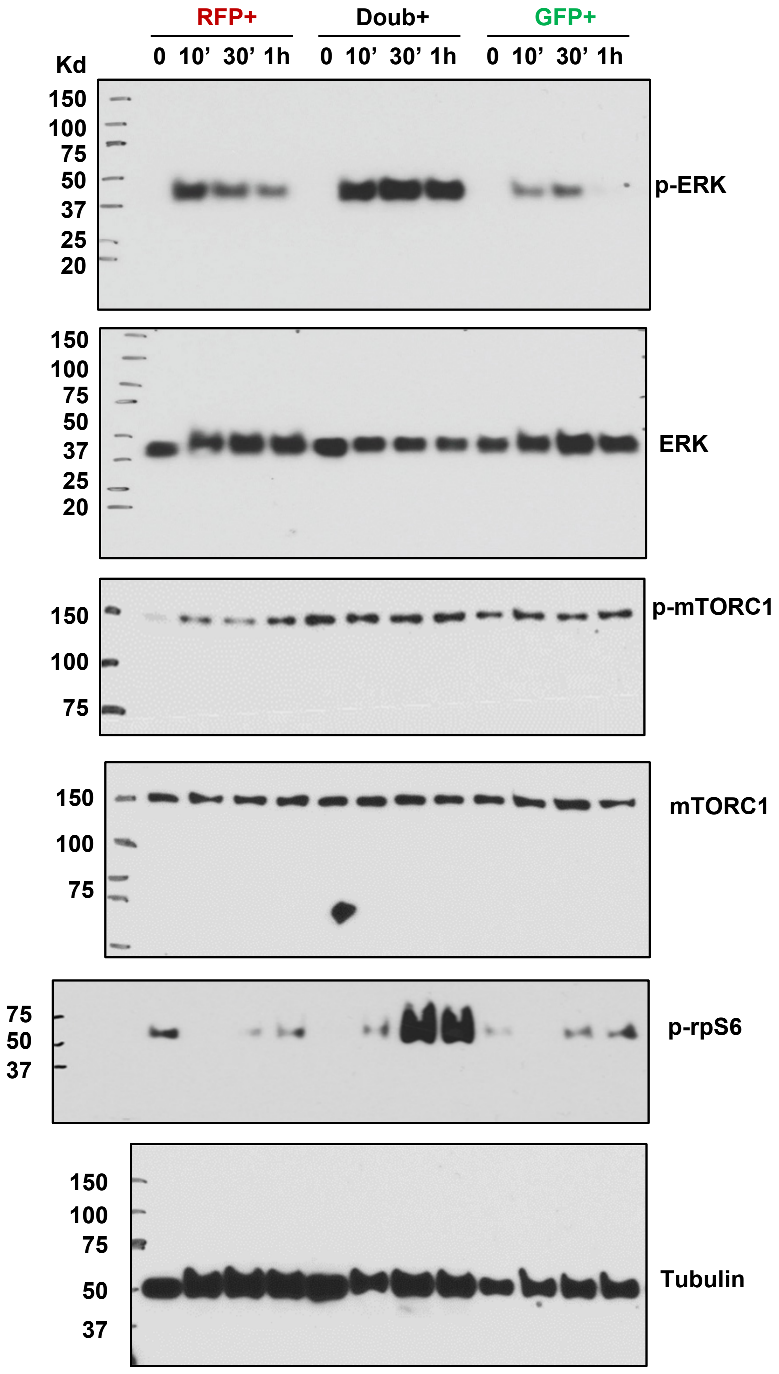

Supplement: Figure 3—source data 2. [file elife-89486-fig3-data2.pdf]
