# Supplementary material for: Targeting ribosome biogenesis as a novel therapeutic approach to overcome EMT-related chemoresistance in breast cancer
Source: eLife. 2024 Sep 11;12:RP89486. doi: 10.7554/eLife.89486 (PMC11390108; doi:10.7554/eLife.89486)

Figure 4B, Western blots of the epithelial marker (E-cad) and mesenchymal markers (Vim and Snail) in Tri-PyMT cells following a 5-day treatment with BMH21 (100nM) and CX5461 (20nM).

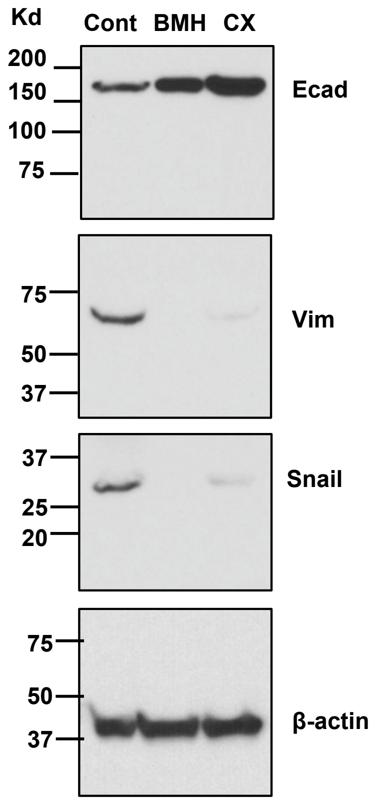

Supplement: Figure 4—source data 2. [file elife-89486-fig4-data2.pdf]
