# Supplementary material for: Targeting ribosome biogenesis as a novel therapeutic approach to overcome EMT-related chemoresistance in breast cancer
Source: eLife. 2024 Sep 11;12:RP89486. doi: 10.7554/eLife.89486 (PMC11390108; doi:10.7554/eLife.89486)

Figure 4-figure supplement 2A, Western blots show the knockdown expression of Rps24 and Rps28 in targeted Tri-PyMT cells.

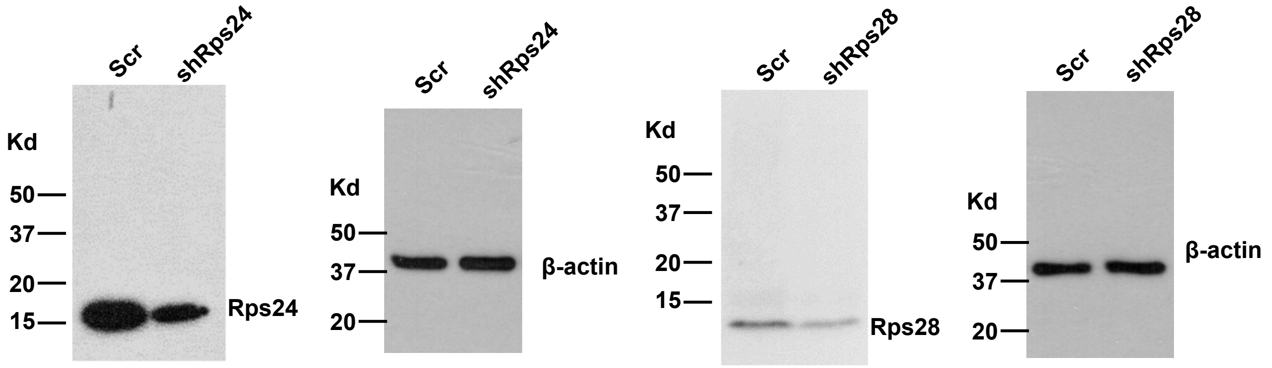

Supplement: Figure 4—figure supplement 2—source data 2. [file elife-89486-fig4-figsupp2-data2.pdf]
